# SELF-DIRECTED CARE IN HOME-BASED LONG-TERM CARE DURING THE PANDEMIC: POLICY AND PRACTICE IMPLICATIONS

**DOI:** 10.1093/geroni/igac059.634

**Published:** 2022-12-20

**Authors:** Carrie Wendel-Hummell, Tracey LaPierre, Darcy Sullivan, Jennifer Babitzke, Lora Swartzendruber, Danielle Olds

**Affiliations:** University of Kansas, Lawrence, Kansas, United States; University of Kansas, Lawrence, Kansas, United States; University of Kansas, Lawrence, Kansas, United States; University of Kansas, Lawrence, Kansas, United States; University of Kansas, Lawrence, Kansas, United States; St. Lukes Hospital, Kansas City, Missouri, United States

## Abstract

The COVID-19 pandemic highlighted strengths and challenges of the self-directed care model for home-based long-term care. We discuss policy and practice implications drawing on interviews with over 50 home-and-community-based-services consumers, caregivers, workers, and providers in Kansas. Low-pay, lack of benefits, rising wages in competing sectors, enhanced unemployment and COVID-19 concerns exacerbated workforce shortages that compromised consumer safety and well-being. The lack of budget authority for self-directed consumers in Kansas limited their ability to address these issues. Furthermore, the self-directed model was excluded from emergency funding sources that would have enhanced pay and benefits for workers, including sick pay for quarantine, pointing to the need for targeted funding. Emergency flexibility allowing paid family caregivers addressed care needs for some but is temporary and should be expanded. In the managed care model, MCOs still kept their capitated payment despite significant unfilled care hours, and thus pay-for-performance incentives need to be revisited.

